# Supplementary material for: Enteroaggregative Escherichia coli in mid-Norway: A prospective, case control study
Source: PLoS One. 2024 Apr 18;19(4):e0301625. doi: 10.1371/journal.pone.0301625 (PMC11025732; doi:10.1371/journal.pone.0301625)
Supplement: S1 Table — (DOCX) [file pone.0301625.s001.docx]

|  | **Diarrhoeal episodes**  **n=9487** | **Healthy controls**  **n=375** |
| --- | --- | --- |
| **Number of pathogens** |  |  |
| 0 | 5655 (59.6%) | 318 (84.8%) |
| 1-5 | 3832 (40.4%) | 57 (15.2%) |
| 1 | 2915 (30.7%) | 52 (13.9%) |
| 2 | 705 (7.4%) | 5 (1.3%) |
| 3 | 149 (1.6%) | 0 |
| 4 | 47 (0.5%) | 0 |
| 5 | 16 (0.2%) | 0 |
| **Type of pathogen** |  |  |
| Any bacterium | 2665 (28.1%) | 49 (13.1%) |
| Any virus | 1380 (14.5%) | 6 (1.6%) |
| Any parasite | 261 (2.8%) | 2 (0.5%) |
|  | | |
