# Supplementary material for: Enteroaggregative Escherichia coli in mid-Norway: A prospective, case control study
Source: PLoS One. 2024 Apr 18;19(4):e0301625. doi: 10.1371/journal.pone.0301625 (PMC11025732; doi:10.1371/journal.pone.0301625)
Supplement: S2 Table — *Travel to more than one continent included in numbers; 22 of the diarrhoeal episodes and 1 of the healthy controls travelled to more than one continent ^Regions as defined by the Statistics Division of The United Nations Secretariat # Prevalence of EAEC in travellers to same region. (DOCX) [file pone.0301625.s002.docx]

| **Travel destination***^ | **Diarrhoeal episodes** | | **Healthy controls** | |
| --- | --- | --- | --- | --- |
|  | All  n = 9487 | EAEC-positive  n = 440 | All  n = 375 | EAEC-positive  n = 8 |
| No travel mentioned | 8116 (85.5%) | 178 (2.2%^#^) | 357 (95.2%) | 8 (2.2%^#^) |
| Any travel destination | 1371 (14.5%) | 262 (19.1%^#^) | 18 (4.8%) | 0 |
| Europe | 594 (6.3%) | 31 (5.2%^#^) | 16 (4.3%) |  |
| Asia | 419 (4.4%) | 104 (24.8%^#^) | 0 |  |
| Africa | 188 (2.0%) | 83 (44.1%^#^) | 0 |  |
| Latin, Central and South America | 118 (1.2%) | 40 (33.6%^#^) | 1 (0.3%) |  |
| Unspecified region | 60 (0.6%) | 7 (11.7%^#^) | 1 (0.3%) |  |
| Northern America | 16 (0.2%) | 0 | 2 (0.5%) |  |
| Oceania | 10 (0.1%) | 2 (20.0%^#^) | 0 |  |
