# Supplementary material for: Enteroaggregative Escherichia coli in mid-Norway: A prospective, case control study
Source: PLoS One. 2024 Apr 18;19(4):e0301625. doi: 10.1371/journal.pone.0301625 (PMC11025732; doi:10.1371/journal.pone.0301625)
Supplement: S3 Table — *Diarrhoeal episodes, ¤EAEC-positive Diarrhoeal episodes, #HC = Healthy controls, %EAEC-positive Healthy controls, &proportion of EAEC-positive Diarrhoeal episodes, +proportion of EAEC-negative Healthy controls. (DOCX) [file pone.0301625.s003.docx]

|  | **Jan** | **Feb** | **Mar** | **Apr** | **May** | **Jun** | **Jul** | **Aug** | **Sep** | **Oct** | **Nov** | **Dec** |
| --- | --- | --- | --- | --- | --- | --- | --- | --- | --- | --- | --- | --- |
| **2017** |  |  |  |  |  |  |  |  |  |  |  |  |
| DE* | - | - | 447 | 321 | 383 | 367 | 379 | 515 | 432 | 395 | 459 | 381 |
| EAEC+DE^¤^ | - | - | 31 | 18 | 13 | 9 | 27 | 36 | 20 | 9 | 9 | 11 |
| HC^#^ | - | - | 1 | 3 | 18 | 25 | 15 | 8 | 7 | 22 | 14 | 13 |
| EAEC+HC^%^ | - | - | 0 | 0 | 1 | 0 | 0 | 1 | 0 | 0 | 0 | 1 |
| **2018** |  |  |  |  |  |  |  |  |  |  |  |  |
| DE* | 399 | 402 | 429 | 432 | 344 | 328 | 356 | 451 | 398 | 407 | 404 | 283 |
| EAEC+DE^¤^ | 23 | 27 | 25 | 14 | 16 | 8 | 17 | 34 | 11 | 17 | 19 | 11 |
| HC^#^ | 23 | 7 | 2 | 0 | 43 | 30 | 9 | 15 | 1 | 33 | 26 | 14 |
| EAEC+HC^%^ | 0 | 0 | 0 | 0 | 1 | 2 | 0 | 0 | 0 | 1 | 1 | 0 |
| **2019** |  |  |  |  |  |  |  |  |  |  |  |  |
| DE* | 399 | 376 | - | - | - | - | - | - | - | - | - | - |
| EAEC+DE^¤^ | 19 | 18 | - | - | - | - | - | - | - | - | - | - |
| HC^#^ | 28 | 18 | - | - | - | - | - | - | - | - | - | - |
| EAEC+HC^%^ | 0 | 0 | - | - | - | - | - | - | - | - | - | - |
| **Total** |  |  |  |  |  |  |  |  |  |  |  |  |
| DE* | 798 | 778 | 876 | 753 | 727 | 695 | 735 | 966 | 830 | 802 | 863 | 664 |
| EAEC+DE^¤^ | 42 | 45 | 56 | 32 | 29 | 17 | 44 | 70 | 31 | 26 | 28 | 22 |
| %EAEC+DE^&^ | 5.3% | 5.8% | 6.4% | 4.2% | 4.0% | 2.4% | 6.0% | 7.2% | 3.7% | 3.2% | 3.2% | 3.3% |
| HC^#^ | 51 | 25 | 3 | 3 | 61 | 55 | 24 | 23 | 8 | 55 | 40 | 27 |
| EAEC+HC^%^ | 0 | 0 | 0 | 0 | 2 | 2 | 0 | 1 | 0 | 1 | 1 | 1 |
| %EAEC+HC^+^ | 0 | 0 | 0 | 0 | 3.3% | 3.6% | 0 | 4.3% | 0 | 1.8% | 2.5% | 3.7% |
