# Supplementary material for: Enteroaggregative Escherichia coli in mid-Norway: A prospective, case control study
Source: PLoS One. 2024 Apr 18;19(4):e0301625. doi: 10.1371/journal.pone.0301625 (PMC11025732; doi:10.1371/journal.pone.0301625)
Supplement: S4 Table — (DOCX) [file pone.0301625.s004.docx]

|  | **EAEC-positive**  **n = 440** | **EAEC-negative**  **n = 9047** |
| --- | --- | --- |
| Female | 239 (54.3%) | 4914 (54.3%) |
| Median age | 30 | 38 |
| Travel history | 262 (59.5%) | 1115 (12.3%) |
| >1 pathogens | 288 (65.5%) | 634 (18.4%) |
