# Supplementary material for: Enteroaggregative Escherichia coli in mid-Norway: A prospective, case control study
Source: PLoS One. 2024 Apr 18;19(4):e0301625. doi: 10.1371/journal.pone.0301625 (PMC11025732; doi:10.1371/journal.pone.0301625)
Supplement: S5 Table — *We only had information on urban or non-urban residency in 233 EAEC-positive with concomitant pathogens, 121 EAEC positive without concomitant pathogens, and 7639 EAEC-negative diarrhoeal episodes ^Only EAEC-positive with concomitant pathogens versus EAEC-negative were significant with p<0.05 in pairwise analyses. (DOCX) [file pone.0301625.s005.docx]

|  | **EAEC-positive with concomitant pathogens n = 288** | **EAEC-positive alone**  **n = 152** | **EAEC-negative**  **n = 9047** | **p-value** |
| --- | --- | --- | --- | --- |
| Female | 154 (53.5%) | 85 (55.9%) | 4914 (54.3%) | p=0.89 |
| Urban residency | 163 (70.0%*) | 77 (64.0%*) | 4539 (59.4%*) | p<0.01^ |
| Median age (years) | 28.5 | 35.5 | 38.0 | p<0.01^ |
| Travel history | 208 (72.2%) | 54 (35.5%) | 1115 (12.3%) | p<0.01 |
| EAEC PCR median CT | 22 | 22 | NA | p=0.59 |
