# Supplementary material for: Enteroaggregative Escherichia coli in mid-Norway: A prospective, case control study
Source: PLoS One. 2024 Apr 18;19(4):e0301625. doi: 10.1371/journal.pone.0301625 (PMC11025732; doi:10.1371/journal.pone.0301625)
Supplement: Attachment — Submitted filename: Review PONE-D-23-26927.docx [file pone.0301625.s006.docx]

Review PONE-D-23-26927

TITLE: *Enteroaggregative Escherichia coli in persons with diarrhoea and healthy controls in mid-Norway: A prospective, case control study*

The study describes the EAEC findings from patients at a university hospital in Mid-Norway with comparisons to healthy subjects.

The findings in the control group are, as the authors themselves comment, less frequently described in a group with no immediate intention to travel; in traveller studies the pre-travel samples have mostly been collected at travel clinics, and those with an intention to travel are also likely to have travelled in the past. However, Bruijnesteijn van Coppenraet et al. have a rather similar study setting in the Netherlands in their 2015 article.

The main problem with the study lies in the control group setting. Nontravellers should not be used as controls: Among asymptomatic travellers, EAEC is found in 16-28% of stool samples (Paschke et al. 2011, Lääveri et al. 2016, Lertsethtakarn-Ketwalha et al. 2018). By contrast, as the authors observe, EAEC is a rare finding in pre-travel stool samples in many traveller studies.

The authors state that nontravellers were defined as not having travel history documented. This does not automatically equal to having no travel history since unless this was actively documented as “no travel history” instead of information on travel history missing i.e. not documented at all.

To my knowledge, the interplay between the various gastrointestinal pathogens has been poorly described in clinical settings particularly among travellers. Furthermore, the association of EAEC with age is not independent from history of travel to low- and middle-income countries; EAEC can be found in stools for several weeks after travel. Therefore, in addition to using nontravellers as controls, I don’t agree with the use of a multivariable regression model used for the identification of the role of EAEC. A more practical approach would have been “only EAEC” vs. “no EAEC” vs “EAEC together with other pathogens. In addition, asymptomatic colonization with Salmonella and even ETEC is relatively common among travellers; this has been described in many studies with control group design (e.g. Paschke et al.2011, Lääveri et al.2016, Lertsethtakarn-Ketwalha et al. 2018). Moreover, the aetiology of diarrhoea among nontravellers is generally considered to be of viral origin although norovirus findings among travellers are more frequent in more recent traveller studies than e.g. 30 years ago.

Unfortunately, due to the issues mentioned above, I cannot recommend accepting this article. However, I encourage the authors to change this into a more descriptive study instead of trying to assess the role of EAEC in diarrhoea; for that purpose they would need to analyze travellers and nontravellers separately and the proportion of travellers in the control group appears too small for statistical analyses.
